# Sensitive Detection of Copper(II) Ions in Water Using Nitrogen-Doped Carbon Quantum Dots Synthesized from Pine Cone Lignin and Lysine

**DOI:** 10.1021/acsomega.6c02895

**Published:** 2026-06-19

**Authors:** Yunus Emre Toprak, Ayben Kilic-Pekgozlu, Soner Çubuk, Sezgin Koray Gülsoy, Elanur Kalkan, Özge Özgürlük, Memet Vezir Kahraman

**Affiliations:** 1 Department of Chemistry, Marmara Universitesi, İstanbul 34722, Turkey; 2 Department of Forest Industry Engineering, Bartın Universitesi, Bartın 74100, Turkey

## Abstract

The accurate and rapid determination of trace copper Cu­(II) ions in environmental water is of great significance for both ecological safety and human health. In this work, a novel, eco-friendly, and highly sensitive fluorescence sensor was developed using carbon dots synthesized from pine cone deep eutectic solvent (DES) lignin and doped with lysine for the selective detection of Cu­(II) ions. The synthesized carbon dots exhibited excellent optical properties. They demonstrated a strong, concentration-dependent fluorescence response to Cu­(II) with a wide linear range of 2.81 × 10^–7^–5.87 × 10^–5^ and a remarkably low limit of detection (LOD) of 2.8 × 10^–8^ mol L^–1^. To validate the practical applicability and reliability of the proposed sensor, it was successfully employed for Cu­(II) determination in highly complex matrices, including various certified reference materials (primary drinking water, NASS-5 seawater, SM-WW1, and SM-WW2 wastewater) and real environmental samples (ground, lake, and tap water). The standard addition method yielded excellent recovery rates ranging from 98.9 to 104.4%, with relative standard deviations (RSDs) strictly below 4.3%. Furthermore, the analytical results were statistically compared with the standard flame atomic absorption spectrometry (FAAS) method at a 95% confidence level, showing exceptional agreement and no significant differences. The proposed lysine-doped carbon dot sensor offers a highly accurate, cost-effective, and highly selective alternative for the routine monitoring of Cu­(II) in diverse and complex environmental water samples, effectively overcoming significant matrix interference.

## Graphical abstract

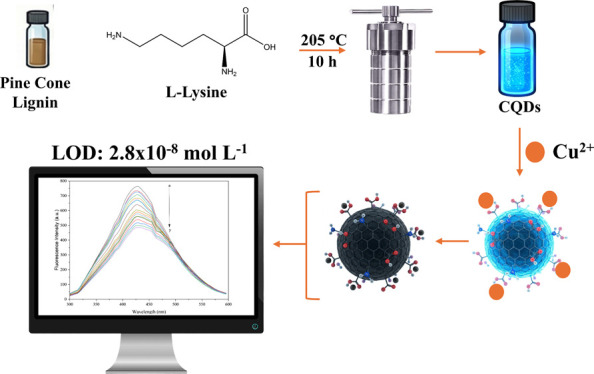

## 1 Introduction

Contamination of aquatic environments due to rapid industrialization and unplanned urbanization has become a critical global environmental and public health challenge. Among various pollutants, heavy metals such as arsenic (As), lead (Pb), mercury (Hg), and cadmium (Cd) are among the priority pollutants due to their nonbiodegradable structures, their tendency to accumulate in living things through the food chain, and the carcinogenic effects they show even at low concentrations. The effective monitoring and removal of these pollutants using conventional treatment and detection methods are often limited by insufficient sensitivity, high costs, and complex processing requirements. Therefore, in recent years, nanotechnology-based materials, especially carbon quantum dots (CQDs), have emerged as a promising alternative for the sensitive detection and removal of heavy metal ions.^1–3^ Alongside CQDs, other novel quantum dot systems, including MXene quantum dots and graphene quantum dots, have also attracted significant attention as highly effective nanomaterial-based sensors.^4–6^

CQDs are carbon-based zero-dimensional nanomaterials, typically smaller than 10 nm. Owing to their high surface area and abundant surface functional groups, they can easily interact with metal ions. Surface groups such as hydroxyl (−OH), carboxyl (−COOH), and amino (−NH_2_) groups can bind heavy metal ions via coordination interactions, enabling sensitive and selective detection even at very low concentrations.^7,8^ In addition, their strong, tunable fluorescence properties make them particularly advantageous for sensor applications. Their low toxicity, good biocompatibility, chemical stability, and ease of surface modification make them well-suited for environmental monitoring and water treatment applications.^2,9,10^

However, the carbon source used in the CQD production plays a decisive role in terms of cost and sustainability. Conventional synthesis methods often rely on expensive or environmentally unsuitable organic precursors. Therefore, in recent years, studies of CQD production from natural, renewable, and low-cost carbon sources have rapidly increased. In this context, lignin has attracted considerable attention as a suitable biomass precursor because of its high carbon content, aromatic structure, and wide availability.^11,12^

Lignin is a three-dimensional amorphous biopolymer with three main units: guaiacyl (G), syringyl (S), and phenol (H). The ratio between these units varies depending on the plant species. Softwoods, such as pine, are composed mainly of guaiacyl (G) units. In contrast, hardwoods, like eucalyptus, contain a mixture of guaiacyl (G) and syringyl (S) units, and grasses, such as wheat, additionally incorporate significant amounts of *p*-hydroxyphenyl (H) units into their lignin structure. Various ether and carbon–carbon bonds link together monolignols. The composition of these groups (S/G/H ratio) and functional groups, such as methoxyl, carboxyl, and phenolic hydroxyl, in lignin facilitates surface functionalization during CQD synthesis.^13,14^

Cones represent the reproductive structures of coniferous trees, characterized by an axis bearing protective scales that enclose the seeds. Although cones are generated annually, they remain largely unutilized in industrial applications. The lignin content of cones ranges from 28 to 48% across species, indicating their potential as a lignin-rich biomass feedstock.^15^ Different biomass sources (e.g., rice husk, coffee grounds, and watermelon peel) have been utilized for CQD production.^16^ More specifically, significant advances have recently been reported regarding the design of biomass-derived CQDs acting as highly sensitive fluorescent probes for Cu­(II) detection.^17–22^

While these pioneering studies have successfully demonstrated the potential of natural carbon sources in sensing applications, achieving high sensitivity alongside a single-step green synthesis from unutilized forestry waste remains a highly desirable goal. Therefore, forest waste, such as pine cones, presents an excellent untapped precursor for CQD production. In this study, cheap and abundant pine cone lignin was processed via a single-step green synthesis method in the presence of lysine to produce highly functional nitrogen-doped CQDs. While hydrothermal synthesis represents an established methodology, this work uniquely integrates the sustainable valorization of pine cone DES-lignin with a targeted lysine doping strategy to specifically address trace Cu­(II) detection. The novelty of this work is highlighted by three main aspects: (1) the sustainable valorization of unutilized pine cone waste biomass into a high-value nanomaterial, promoting a circular economy; (2) the achievement of a competitive limit of detection (LOD of 2.8 × 10^–8^ mol L^–1^) for Cu­(II), which is highly competitive with existing biomass-derived sensors published recently; and (3) the sensor’s excellent matrix tolerance, enabling highly accurate and reliable trace detection even in highly complex real-world matrices, such as certified seawater (NASS-5) and wastewater. This robust validation against diverse certified reference materials (CRMs) demonstrates a level of practical applicability that distinguishes this research from standard proof-of-concept studies reported in the literature. The integration of lysine provides nitrogen-rich surface functionalities that are structurally well-suited to serve as a robust and sustainable fluorescence sensor platform.

## 2 Experimental Detail

### 2.1 Materials and Instrumentation

Chemical reagents were obtained from Sigma-Aldrich (St. Louis, MO) and used without purification. All aqueous solutions were prepared by using ultrapure water (Milli-Q).

To assess the synthesized CQDs, multiple characterization tools were utilized. Optical evaluations included UV–vis (Shimadzu UV-1800), PL (Varian Cary Eclipse), and FTIR (Spectrum 100) spectroscopy. Particle size was determined via dynamic light scattering (DLS) utilizing a Malvern Zetasizer and Brookhaven 90Plus equipment. The crystal structure was probed using a Philips XRD system with Cu Kα radiation. Furthermore, the morphology and surface chemical states were analyzed using a Thermo Scientific Quattro S SEM/STEM and a Thermo Fisher Scientific K-Alpha X-ray photoelectron spectroscopy (XPS), respectively.

Choline chloride (ChCl) and lactic acid (LA) were combined in a molar ratio of 1:2 to form the DES mixture. A clear solution was generated after the mixture was heated to 70 °C for 30 min while being continuously stirred. To minimize moisture absorption, it was then cooled to room temperature in a glass desiccator.

*Pinus pinea* L. (Stone pine) cones were used as a lignocellulosic raw material source for lignin. Cone samples were collected from İzmir-Türkiye. The samples were run through a Retsch AS 200 sieve shaker after being ground in a Wiley mill. DES-lignin was extracted by further processing using 40–80 mesh fractions.

### 2.2 Isolation of DES-Lignin from the Cones of Stone Pine

The DES treatments of cone samples were done in a 2 L autoclave bottle using a Hirayama HV-110 autoclave. Cone samples (dry mass 100 g) were placed in an autoclave bottle, and DES (1000 g) was added. The autoclave bottle was left to react at 121 °C for 3 h. After treatment, 500 mL of ethanol was added to the autoclave bottle. Vacuum filtration, using a Buchner funnel and a Macherey-Nagel MN 751/60 filter paper, separated the solid from the liquid. The resultant solid residue was washed with anhydrous ethanol multiple times until the filtrate was colorless. The filter paper was dried at 105 °C for 6 h. The filtrate was evaporated at 80 °C in a rotary evaporator to remove ethanol, and then, 10 volumes of distilled water were added to precipitate lignin. After 48 h of precipitation, the precipitated lignin samples were filtered through Whatman no. 42 filter paper and washed with a 1:9 (v/v) ethanol/distilled water. Finally, the DES-lignin samples were dried in a freeze-dryer. The dried lignin was placed in a sample vial and kept in a desiccator until further analysis.

### 2.3 N-Doped Carbon Quantum Dot Synthesis

Nitrogen-doped carbon quantum dots (CQD) were synthesized via a facile one-pot hydrothermal carbonization method, adapted from prevailing approaches in the literature. In a typical procedure, pine cone DES-lignin (1 g), serving as the biomass carbon precursor, and l-lysine (1 g), providing the nitrogen dopant, were dispersed in 15 mL of distilled water (DI). The mixture was magnetically stirred until a homogeneous suspension was obtained. This precursor mixture was then transferred into a 25 mL Teflon-lined stainless-steel autoclave. The sealed vessel was subsequently subjected to solvothermal treatment in a muffle furnace maintained at 210 °C for 10 h. Following the reaction, the autoclave was allowed to cool naturally to ambient temperature. The resulting dark yellow solution exhibited distinct fluorescence under UV light, indicating the successful formation of CQD. A rigorous purification cascade was employed to isolate the CQD from unreacted precursors and large carbonaceous aggregates. The crude solution was first centrifuged at 12,000 rpm for 16 min to precipitate large particles. The supernatant was carefully collected and filtered through a 0.22 μm membrane filter. The filtrate was dried in vacuo to remove the ethanol solvent. The obtained solid was redispersed in deionized water and subjected to extensive dialysis (MWCO: 1 kDa) against fresh deionized water for 24 h to remove small molecules and ions. Finally, the purified CQD solution was freeze-dried to yield a solid powder, which was stored for subsequent characterization and application (Scheme 1).

**Scheme 1:**
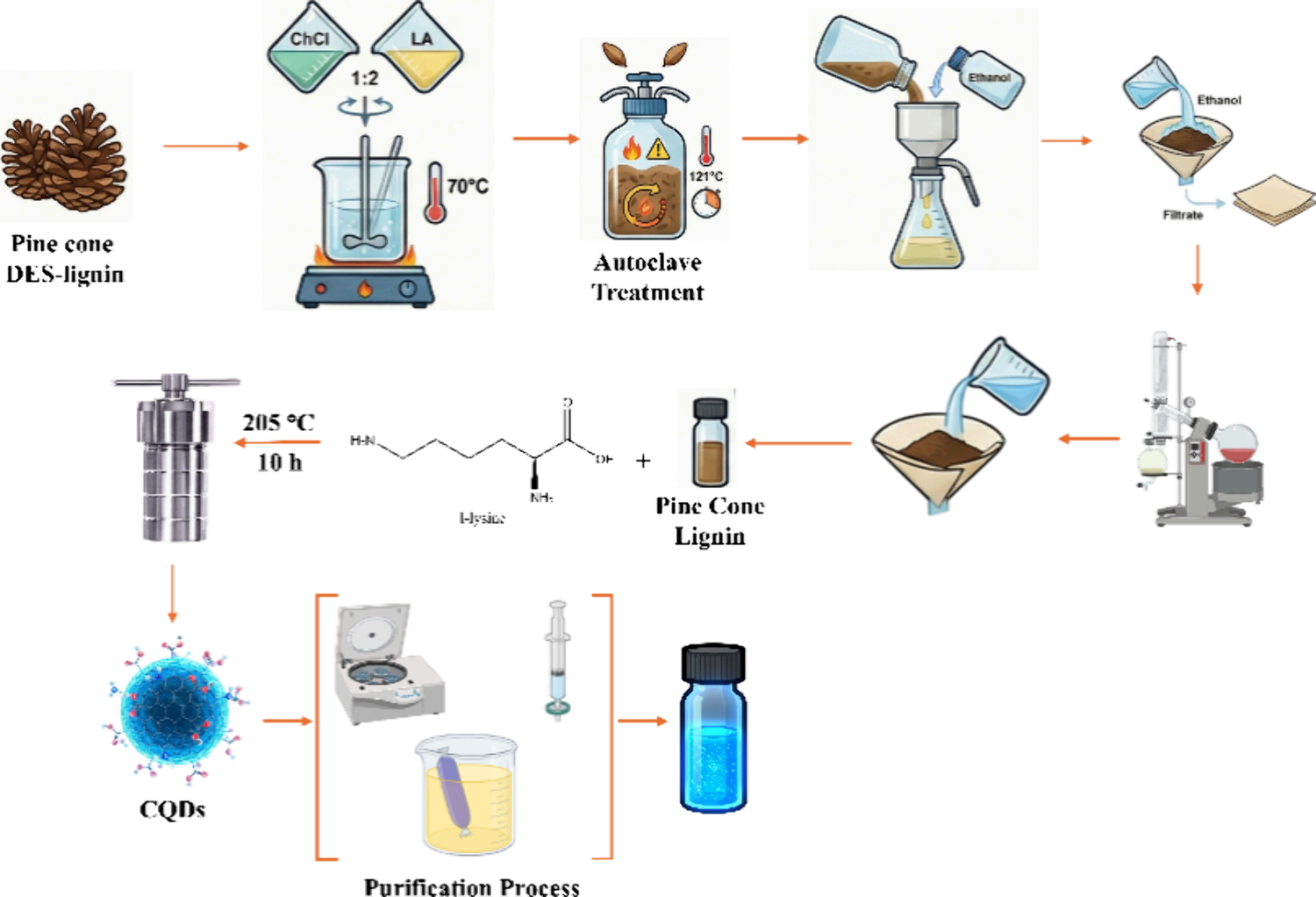
Schematic Representation of the Extraction of DES-Lignin from Pine Cones and the Subsequent Hydrothermal Synthesis and Purification of Nitrogen-Doped CQDs

### 2.4 Measurement of the Quantum Yield

The quantum yield of the prepared CQDs was evaluated using a comparative method. Quinine sulfate in sulfuric acid (reference QY = 54% at 360 nm excitation; refractive index = 1.33) was utilized as the benchmark. Measurements for the CQDs were performed in an aqueous solution (refractive index = 1.33). The relative QY values were derived using the following relationship:

$$\phi_{\mathrm{CD}}=\phi_{\mathrm{QS}}\times ({Ι}_{\mathrm{CD}}/{Ι}_{\mathrm{QS}})\times (\eta_{{\mathrm{CD}}^{2}}/\eta_{{\mathrm{QS}}^{2}})$$

where ϕ, *I*, and η correspond to the quantum yield, integrated photoluminescence intensity, and solvent refractive index, respectively.

**Figure 1:**
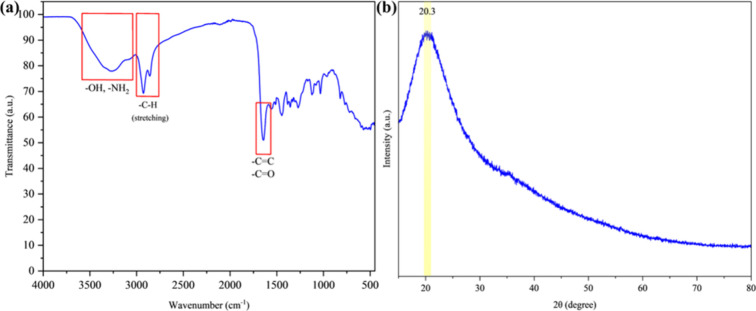
Structural characterization of the CQDs derived from pine cone DES-lignin and lysine: (a) FTIR spectrum revealing the surface functional groups; (b) XRD pattern indicating the amorphous carbon structure.

**Figure 2:**
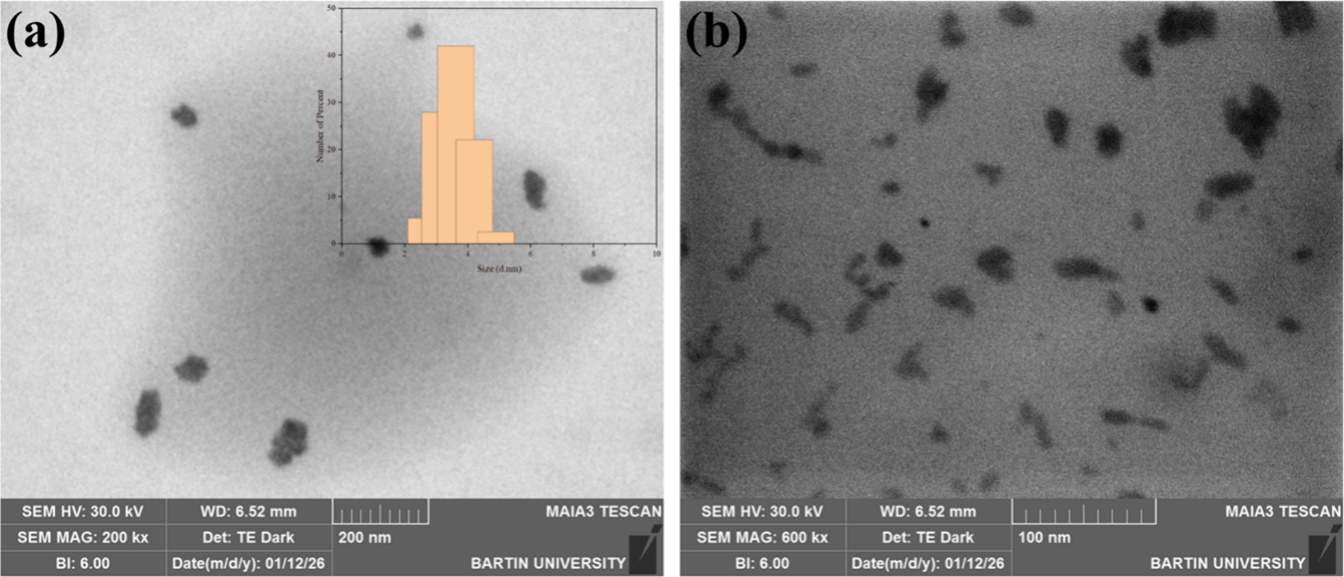
Morphological characterization and size distribution of pine cone DES-lignin/lysine-derived CQDs. (a) STEM micrograph illustrating the quasi-spherical morphology and uniform dispersion of the nanoparticles (inset: hydrodynamic size distribution measured by DLS). (b) High-magnification STEM image revealing surface details and tendencies toward particle aggregation.

**Figure 3:**
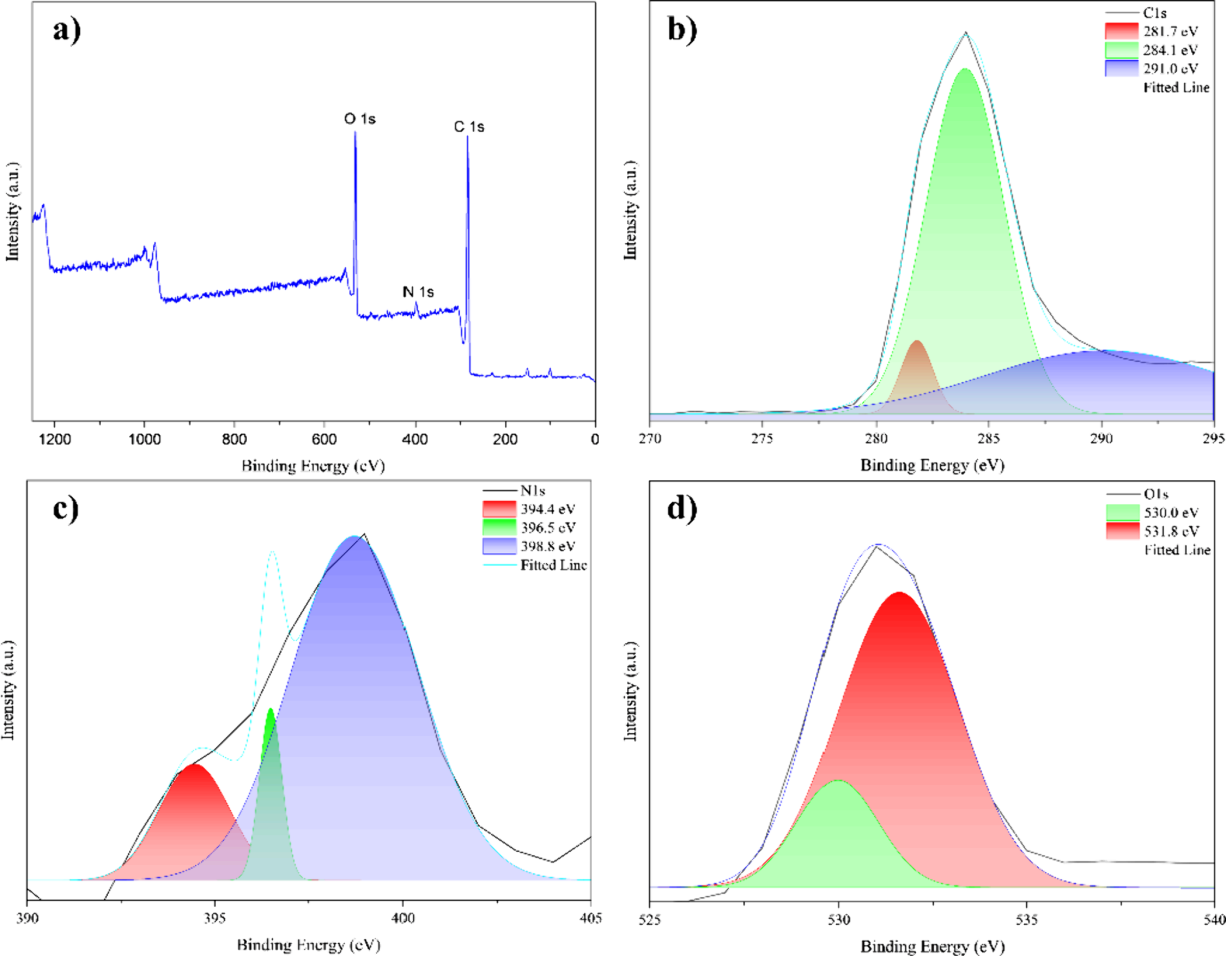
XPS analysis results show the surface elemental composition of pine cone DES-lignin/lysine-based CQDs: (a) full survey scan spectrum, and high-resolution deconvoluted spectra of (b) C 1s, (c) N 1s, and (d) O 1s regions.

### 2.5 Cu­(II) Ion Sensor Detection Procedure

Fluorescence titration was utilized to investigate the Cu­(II) sensing performance of CQDs. Briefly, calculated aliquots of a Cu­(II) stock were added to 3 mL of a CQD dispersion (0.5 mg mL^–1^) in a quartz cuvette. After thorough mixing, the emission spectra were monitored at an excitation of 347 nm to track intensity variations. The visual quenching of the CQD solutions upon copper addition was also photographed under a 365 nm UV light in a dark environment (Figure 4).

**Figure 4:**
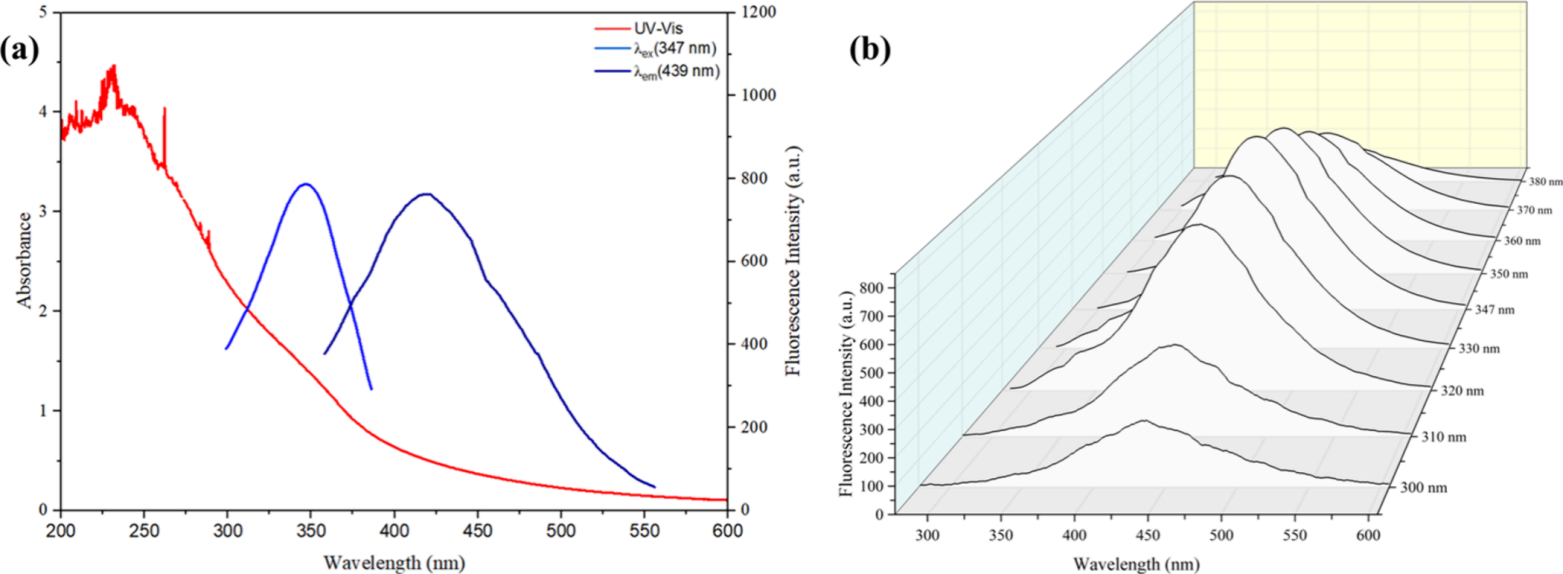
Optical properties of the synthesized CQDs: (a) UV–vis absorption spectrum (red line) overlaid with the excitation (λ_ex_ = 347 nm) and emission (λ_em_ = 439 nm) fluorescence spectra; (b) PL emission spectra recorded under progressively increasing excitation wavelengths (from 300 to 380 nm), demonstrating excitation-dependent emission behavior.

## 3 Results and Discussion

### 3.1 Characterization of CQDs

To comprehensively understand the physicochemical properties of synthesized nanomaterials, CQDs have been systematically characterized using a range of analytical techniques. While the morphology and particle size distribution were examined with STEM and DLS, crystal phase, elemental composition, and surface chemical states were investigated in detail using XRD, FTIR, and XPS, respectively. Furthermore, optical behaviors, including light absorption and excitation-dependent emission characteristics, have been elucidated using UV–vis and PL spectroscopy. The surface chemistry and crystalline phase of the as-synthesized CQDs were investigated using FTIR and XRD analyses, respectively (Figure 1). Figure 1a displays the FTIR spectrum, which provides insight into the successful functionalization of the carbon network. The broad and intense absorption band appearing in the range of 3000–3500 cm^–1^ is attributed to the stretching vibrations of O–H and N–H bonds. The presence of these hydrophilic groups originates from the hydrolysis of lignocellulose and the amine groups of lysine, imparting excellent water solubility to the CQDs. The peaks observed between 2800 and 2900 cm^–1^ correspond to the C–H stretching vibrations of alkyl chains. Furthermore, the characteristic absorption peak centered around 1600–1700 cm^–1^ signifies the existence of C=O (carbonyl/amide) and C=C stretching vibrations of the aromatic sp^2^ carbon domains. This confirms that the hydrothermal treatment successfully carbonized the precursors while retaining essential surface functional groups. In the fingerprint region (<1500 cm^–1^), various bending vibrations (e.g., C–N and C–O) further corroborate the incorporation of nitrogen into the carbon framework.

The crystal structure of the CQDs was analyzed by XRD, as shown in Figure 1b. The pattern exhibits a single broad diffraction peak centered at 2θ = 20.3 °. This broad halo is characteristic of amorphous carbon structures and corresponds to a disordered sp^2^ carbon framework. The absence of sharp diffraction peaks indicates a low degree of graphitization and small particle size, which effectively prevents the long-range stacking of graphite layers. The calculated interlayer spacing (*d*-spacing) is likely larger than that of bulk graphite (0.34 nm), attributed to the introduction of oxygen- and nitrogen-containing functional groups that expand the interlayer distance.

For the dimensional analysis of the synthesized CQDs, the 100 nm scale high-resolution STEM image in Figure 2b was used as the reference. Random particle measurements (*n* = 100) using the ImageJ image-on-image software show that CQDs range from 2.5 to 8.0 nm. The size distribution of the particles conforms to the Gaussian curve, and the calculated average particle diameter is 4.8 ± 1.2 nm. This value confirms that the synthesized material is small enough to exhibit quantum confinement. When the general view of Figure 2a (500 nm scale) was examined, it was observed that nanoparticles formed spherical aggregates or “coffee ring”-like structures of 50–200 nm in size to minimize the surface energy during solvent evaporation, but the actual quantum dots (3–8 nm) were preserved in these structures. Although the individual lattice fringes are not completely resolved in the current STEM magnification, the structural nature of the carbon core—comprising an amorphous framework with disordered sp^2^ domains—is thoroughly corroborated by the complementary XRD and XPS analyses discussed above.

The average hydrodynamic diameter obtained by DLS measurements (Figure 2a, internal graph) was approximately 12 nm. This difference between the average diameter in the dry state (∼4.8 nm) was determined by STEM and the DLS result (∼12 nm). It has been attributed to the thickness of the hydration layer formed by the hydrophilic groups (−OH and −NH_2_) on the surface of the CQD with water molecules and to the fact that DLS is more sensitive to aggregated particles. Although the exact zeta potential could not be measured due to instrumental module limitations, the exceptional colloidal stability of the synthesized CQDs in aqueous media is firmly supported by their surface chemistry. The abundant highly hydrophilic surface functional groups (such as −OH, −NH_2_, and −COOH), which were comprehensively confirmed by FTIR and XPS analyses, provide strong electrostatic and steric stabilization. Empirically, the CQD aqueous dispersions exhibited excellent long-term stability, remaining perfectly homogeneous and free of any visible aggregation or precipitation even after several months of storage at room temperature.

The surface chemical composition and elemental states of the pine cone DES-lignin/lysine-based CQDs were investigated by using XPS. The full survey scan spectrum (Figure 3a) exhibits three dominant peaks corresponding to C 1s, N 1s, and O 1s at binding energies of approximately 284, 399, and 532 eV, respectively. The absence of any other elemental peaks confirms the high purity of the synthesized CQDs and the successful incorporation of nitrogen into the carbon framework.

To further elucidate the specific functional groups and chemical bonding states, high-resolution XPS spectra of C 1s, N 1s, and O 1s were deconvoluted. The high-resolution C 1s spectrum (Figure 3b) is resolved into three distinctive peaks centered at 284.8, 286.2, and 288.5 eV. These peaks are attributed to the sp^2^/sp^3^-hybridized carbon (C–C/C=C) forming the graphitic core, carbon bonded to nitrogen or oxygen (C–N/C-O) indicating surface functionalization, and carbonyl/carboxyl carbon (C=O/O–C=O), respectively.

The successful nitrogen doping is evident in the high-resolution N 1s spectrum (Figure 3c), which can be fitted with three peaks at 398.5, 399.8, and 401.2 eV. These components are characteristic of pyridinic-N, pyrrolic-N, and graphitic-N, respectively. The presence of these diverse nitrogen configurations confirms that lysine effectively acts as a nitrogen dopant. Pyridinic and pyrrolic nitrogen atoms are known to introduce active coordination sites and defect states, which are crucial for enhancing the fluorescence quantum yield and providing active sites for selective-target sensing. Furthermore, the high-resolution O 1s spectrum (Figure 3d) presents two peaks at 531.5 and 532.8 eV, which are assigned to the oxygen atoms in carbonyl (C=O) and hydroxyl/epoxy (C–OH/C–O–C) groups, respectively. The abundance of these oxygen-containing functional groups endows the synthesized CQDs with excellent hydrophilicity, ensuring a superior colloidal stability in aqueous environments.

The optical properties of the synthesized CQDs were studied using UV–vis absorption and photoluminescence spectroscopy (Figure 4). As seen in Figure 4a, the absorption spectrum (red line) exhibits a marked absorption in the UV region. The strong absorption peak in the 200–250 nm range is attributed to the aromatic π–π* electronic transition of sp^2^-hybridized carbon domains; the shoulder observed around 300 nm indicates n−π* transitions of functional groups such as C=O or C–N. These results confirm that the carbon structure is successfully functionalized with nitrogen atoms. When the photoluminescence properties of CQDs were examined (Figure 4a), it was seen that when the material was excited at a wavelength of 347 nm (excitation), it reached its maximum emission intensity at 439 nm and exhibited a strong blue glow. This observed Stokes shift is due to radiative recombination of photoexcited electron-vacancy pairs. Figure 4b details the excitation wavelength-dependent emission behavior of CQDs. It was observed that when the excitation wavelength is increased from 300 to 380 nm, the emission peak intensity first increases and then decreases, reaching a maximum at 347 nm excitation. Furthermore, it is noteworthy that emission peaks shift to longer wavelengths as the excitation wavelength increases. This behavior, characteristic of carbon quantum dots, can be explained by polydispersity in the particle size distribution, arising from surface defects and the presence of different surface energy traps. This suggests that the energy levels generated by functional groups (e.g., −NH_2_, −COOH) on the surface of the CQD contribute to its multicolored emission. The fluorescence quantum yield (QY) of the synthesized pine cone DES-lignin/lysine-derived CQDs was found to be 21.8% (calculated as detailed in Section 2.4) using the comparative method with quinine sulfate as the reference standard. This significant quantum yield is mainly due to the effective nitrogen doping process. The incorporation of nitrogen atoms into the carbon framework introduces new surface defect states and effectively passivates surface traps. This facilitates the radiative recombination of electron–hole pairs, thereby significantly enhancing the overall fluorescence efficiency of the nanoparticles. The specific role of lysine as a nitrogen dopant is crucial for the sensor’s performance. Lysine was strategically selected due to its extra ε-amino group on the side chain, which provides a higher density of primary amine functionalities on the CQD surface compared to other amino acids. The presence of pyridinic-N and amino-N species (at ∼399.5 and ∼401.2 eV), confirmed by the N 1s spectrum, provides specific coordination sites. These nitrogen-containing groups, together with surface carboxylate functionalities, act as multidentate chelating agents that directly coordinate with the vacant d-orbitals of Cu­(II) ions. This synergistic effect not only passivates surface defects, increasing the quantum yield to 21.8%, but also creates a highly selective “binding pocket” that favors Cu­(II) over other transition metals, which is theoretically expected to enhance both sensitivity and selectivity by providing specific coordination sites.

### 3.2 Optimization of Analysis Conditions and Analytical Performance

The critical role of nitrogen doping (derived from lysine) in enhancing the sensor’s performance is supported by both chemical characterization and literature precedents. As confirmed by high-resolution N 1s XPS analysis, lysine serves as a nitrogen source that successfully incorporates pyridinic, pyrrolic, and graphitic nitrogen atoms into the carbon framework. These nitrogen species introduce active coordination sites that are anticipated to facilitate the affinity of the CQDs toward Cu­(II) ions, consistent with the established literature on nitrogen-doped systems. Furthermore, according to the comparative quantum yield (QY) analysis, the achieved QY of 21.8% is notably higher than that of many nondoped biomass-derived CQDs, which typically exhibit QY values below 5–10%. This enhancement is attributed to the passivation of surface defects by nitrogen atoms, which promotes radiative recombination and provides more effective binding motifs for selective Cu­(II) sensing. The high selectivity toward Cu­(II) is primarily attributed to the unique coordination environment provided by the lysine-derived nitrogen sites. These functionalities offer a high affinity for Cu­(II) ions through stable ground-state complex formation, which is more electronically and sterically favorable than that for other potentially interfering metal ions. The fluorescent response (*I*_0_–*I*) increased significantly as the pH increased from 1.0 to 7.0, reaching a maximum at pH 7.0. At lower pH values, the protonation of functional groups (e.g., carboxyl or amino groups) on the probe surface likely hindered coordination with Cu­(II) ions because of electrostatic repulsion and the competitive binding of H^+^ ions. Conversely, at pH values greater than 7.0, a decline in the response was observed, which can be attributed to the formation of copper hydroxide (Cu­(OH)_2_) precipitates or hydroxo complexes, reducing the availability of free Cu­(II) ions for interaction with the sensor. Therefore, pH 7.0 was selected as the optimal condition, and all subsequent analytical measurements, including the construction of the calibration curve, were performed at this optimized pH (Figure 5a).

**Figure 5:**
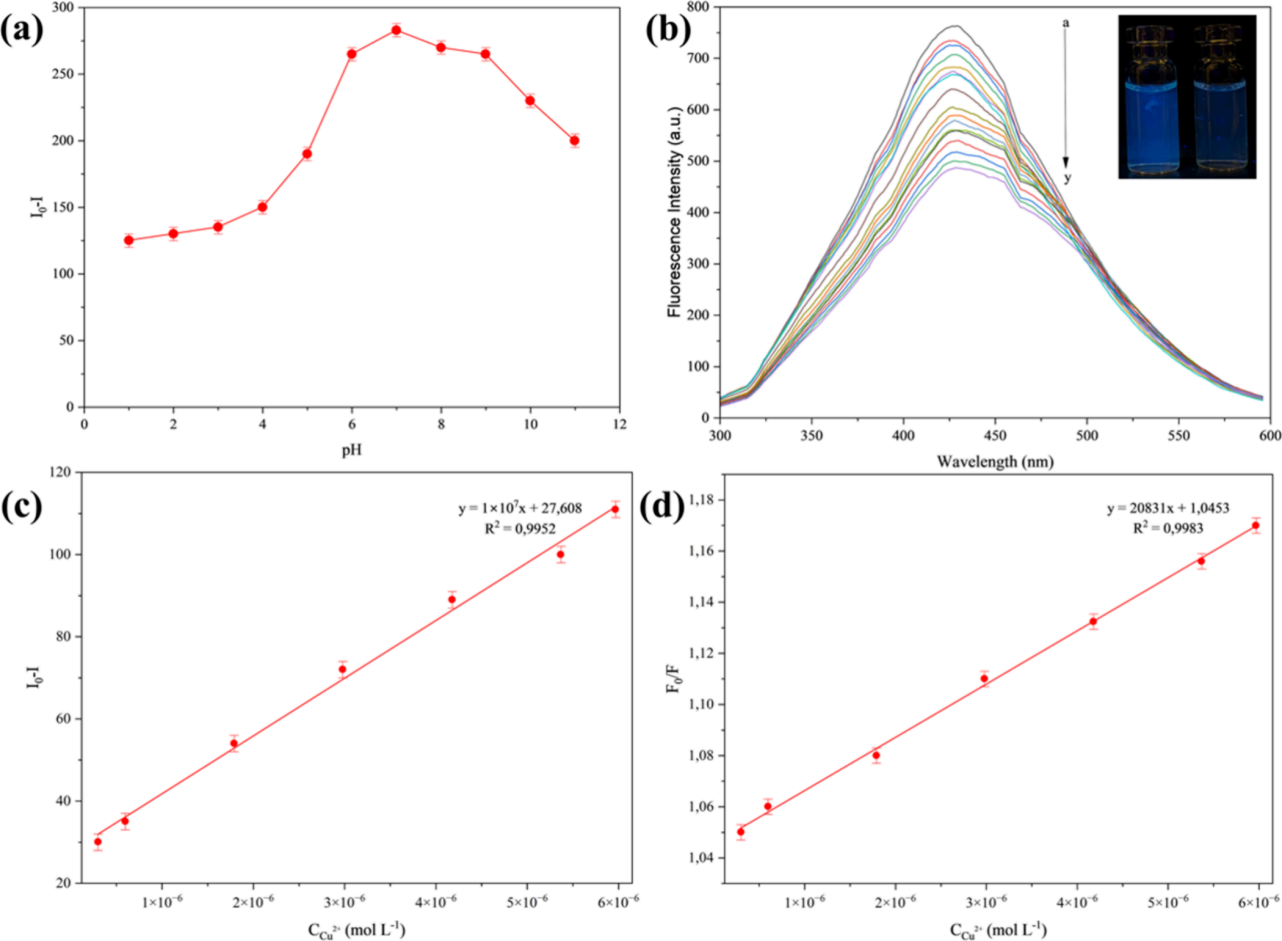
Optimization and sensing performance of the CQD-based copper sensor: (a) effect of pH on the fluorescence quenching efficiency of the sensor, (b) fluorescence emission spectra of CQDs upon the addition of various Cu­(II) concentrations (2.81 × 10^–7^–5.87 × 10^–5^), (c) linear relationship between fluorescence intensity change and Cu­(II) concentration, and (d) Stern–Volmer plot (*F*_0_/*F* vs concentration) elucidating the quenching mechanism.

The quantitative detection ability of the synthesized probe toward Cu­(II) ions was systematically examined by monitoring changes in fluorescence intensity as different concentrations of Cu­(II) were added. As shown in Figure 5b, the probe’s fluorescence emission consistently and significantly decreased with each addition of Cu­(II) ions. The Cu­(II) concentration ranged from a minimum of 2.81 × 10^–7^ M to a maximum of 5.87 × 10^–5^ M. The lack of any noticeable shift in the emission wavelength indicates that the fluorophore’s structure remained intact and that the interaction was mainly driven by stable complex formation or charge transfer mechanisms.

The limit of detection (LOD) for the proposed sensor was calculated using the standard formula LOD = 3σ/S. In this equation, σ represents the standard deviation of the fluorescence intensity from multiple measurements of the blank solution (without Cu­(II), *n* = 10), and S is the slope of the linear calibration curve (1 × 10^7^). Utilizing this method, the LOD was determined to be 2.8 × 10^–8^ mol L^–1^, further confirming the exceptional sensitivity of the pine cone-derived CQDs for trace Cu­(II) detection. Additionally, the limit of quantification (LOQ) was calculated using the formula LOQ = 10σ/S. The LOQ of the proposed sensor was determined to be 9.33 × 10^–8^ mol L^–1^, indicating excellent quantitative capability at trace levels.

To assess the sensor’s analytical performance, the relationship between the change in fluorescence intensity (*I*_0_–*I*) and Cu­(II) concentration was examined (Figure 5c). A clear linear correlation was observed across the studied concentration range, described by the regression equation *y* = 1 × 107*x* + 27.608, with a high determination coefficient (*R* ^2^ = 0.9952). This strong linearity confirms the sensor’s capability for accurate quantitative analysis of copper ions in aqueous solutions. Additionally, the quenching efficiency was assessed using the Stern–Volmer equation (*F*_0_/*F* = 1 + *K*_sv_ [*Q*]), as shown in Figure 5d. The plot demonstrated excellent linearity (*R* ^2^ = 0.9983), indicating that a single quenching mechanism dominates within this range. The calculated Stern–Volmer constant (*K*_sv_) was 2.08 × 10^4^ M^–1^, reflecting the probe’s high sensitivity and strong affinity for Cu^2+^ ions. Based on this excellent linearity and the strong coordinating ability of the surface functional groups (e.g., −OH and −NH_2_), the interaction is predominantly consistent with a static quenching mechanism. This involves the formation of a stable, nonfluorescent ground-state complex between the Cu^2+^ ions and the CQDs. Furthermore, the proximity of the bound Cu^2+^ ions to the CQD surface highly facilitates a photoinduced electron transfer (PET) or nonradiative charge transfer process from the excited state of the CQDs to the unfilled d-orbitals of the Cu^2+^ ions, which fundamentally drives the observed efficient fluorescence quenching.

Although a linear Stern–Volmer plot indicates a single quenching mechanism, it does not distinguish between static and dynamic processes. To further evaluate the static quenching mechanism, the bimolecular quenching rate constant (kq) was calculated using the equation *K*_sv_ = *k*_q_τ_0_. Assuming the average fluorescence lifetime (τ_0_) of typical carbon dots to be around 5 ns, the apparent *k*_q_ value for our system was calculated as approximately 4.16 × 10^12^ M^–1^ s^–1^. This value is orders of magnitude higher than the maximum diffusion-controlled collision rate constant in water (2.0 × 10^10^ M^–1^ s^–1^). This exceptionally high *k*_q_ value physically precludes a purely dynamic (collisional) process. It indicates that the fluorescence quenching is consistent with a static quenching mechanism, involving the formation of a stable, nonfluorescent CQD–Cu^2+^ complex at the ground state.

### 3.3 Cu­(II) Ion Selectivity Study

To establish the practical applicability and high specificity of the proposed chemosensor, its selectivity toward Cu­(II) was rigorously evaluated against a comprehensive panel of potential interfering metal ions, including Fe^2+^, Hg^2+^, Au^3+^, Pb^2+^, As^3+^, BO_3_^3–^, Ni^2+^, Cd^2+^, Cr^3+^, Na^+^, Mg^2+^, Ti^4+^, V^5+^, Ag^+^, Al^3+^, Zn^2+^, Sn^2+^, and Mn^2+^. The selectivity profiles are quantitatively presented in Figure 6. As is clear from the bar diagram, the probe exhibited a remarkably distinct and high response solely to the presence of Cu­(II) ions, resulting in a substantial signal change (*I*_0_–*I*) exceeding 280. In sharp contrast, all other tested cations, even at the same concentration, induced negligible signal variations (*I*_0_–*I* < 20) under identical experimental conditions. This pronounced and specific response to Cu­(II) compared with the minimal effects observed with other biologically and environmentally relevant metal ions unequivocally demonstrates the probe’s excellent selectivity. This exceptional specificity is likely due to a highly favorable, specific coordination geometry between the Cu­(II) ion and the probe’s binding motif, suggesting significant potential as a reliable tool for copper detection in complex sample matrices with minimal cross-reactivity.

**Figure 6:**
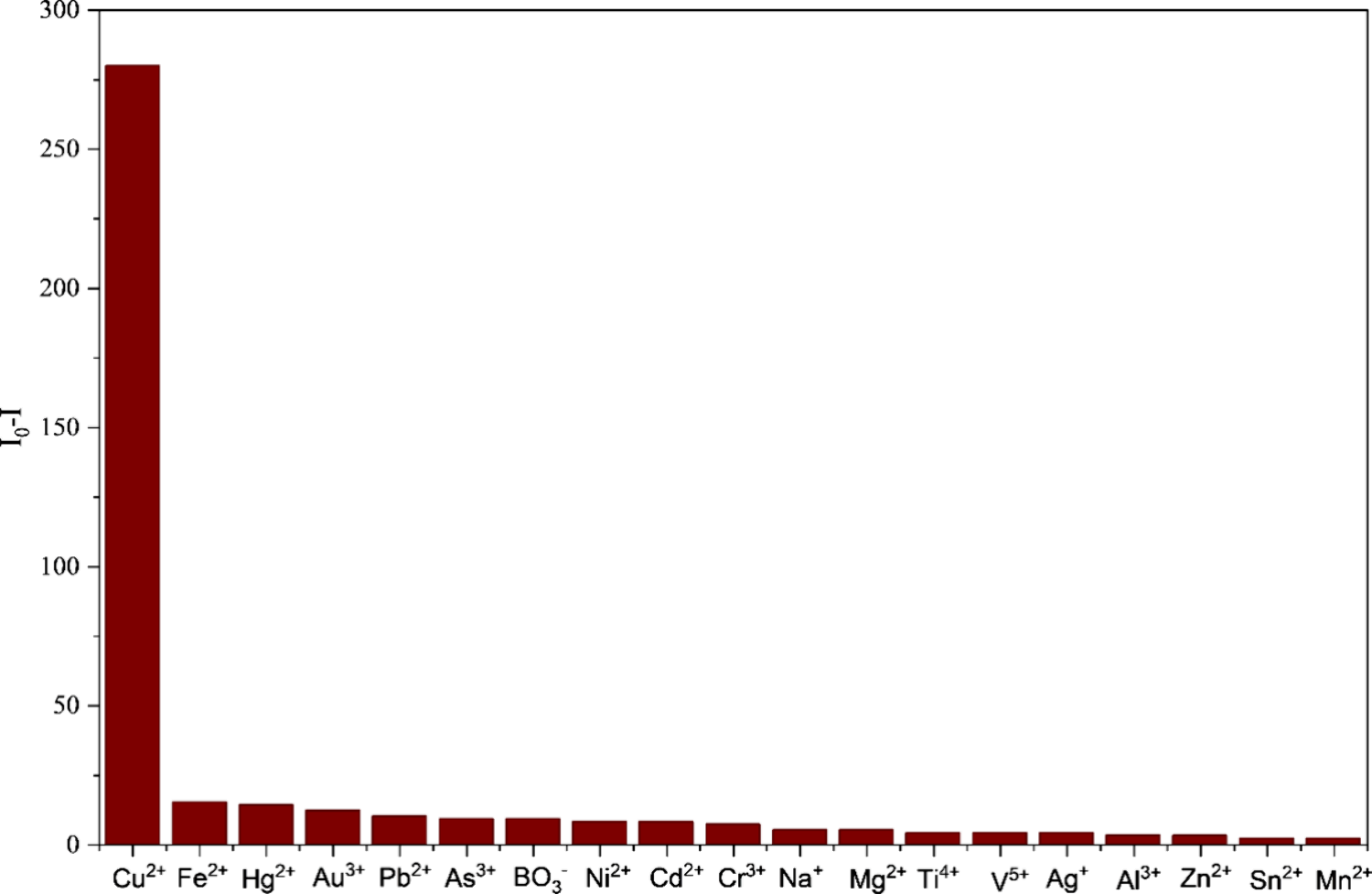
Selectivity histogram of the probe toward Cu­(II) ions compared to various other potential interfering metal ions. The bars represent the magnitude of the signal change (*I*_0_–*I*).

When positioned against the recent CQD-based sensor literature, our pine cone-derived N-doped system exhibits distinct advantages. While sulfur/nitrogen codoped CQDs often target Hg­(II) ions due to the strong affinity of sulfur for mercury, our single-doping strategy with lysine creates a nitrogen-rich surface specifically optimized for the coordination geometry of Cu­(II) [3,17–19]. Furthermore, compared to other biomass-derived systems (e.g., those using fruit peels or agricultural waste), our DES-lignin-based precursor provides a more stable graphitic carbon core, as evidenced by high matrix tolerance in complex NASS-5 seawater matrices. Unlike many Cu­(II) probes that operate over narrow pH ranges, the present sensor maintains high sensitivity across a broad physiological and environmental pH window (pH 5.0–9.0), bridging the gap between sustainable materials and high-performance analytical tools.

### 3.4 Determination of Cu­(II) in Real Samples

To evaluate the practical applicability and accuracy of the developed method for complex matrices, Cu­(II) was determined in various certified reference materials (CRMs), including a primary drinking water CRM, NASS-5, and SM-WW1 and SM-WW2. The standard addition method was employed to evaluate and mitigate the potential matrix effects. The analytical results and recovery values are listed in Table 1.

**Table 1:** Determination of Cu­(II) in Certified Reference Materials and Evaluation of Recovery Rates by the Proposed Method (Mean ± SD, *n* = 3)^a^

sample	certificate value (mol L^–1^)	added Cu(II) concentration (mol L^–1^)	current study (mol L^–1^)	RSD (%)	recovery (%)
**primary drinking water CRM**	NC		ND		
	NC	1.00 × 10^–6^	(1.03 ± 0.03) × 10^–6^	3.21	103.1
	NC	2.00 × 10^–6^	(2.04 ± 0.04) × 10^–6^	2.13	102.2
	NC	3.00 × 10^–6^	(3.13 ± 0.13) × 10^–6^	4.10	104.2
**NASS-5CRM**	(4.67 ± 0.72) × 10^–9^		(4.63 ± 0.16) × 10^–9^	3.55	
	(4.67 ± 0.72) × 10^–9^	5.00 × 10^–9^	(9.76 ± 0.28) × 10^–9^	2.84	102.6
	(4.67 ± 0.72) × 10^–9^	1.00 × 10^–8^	(1.50 ± 0.06) × 10^–8^	4.29	103.7
	(4.67 ± 0.72) × 10^–9^	1.50 × 10^–8^	(1.95 ± 0.04) × 10^–8^	1.90	98.9
**SM-WW1**	(6.29 ± 0.03) × 10^–6^		(6.28 ± 0.15) × 10^–6^	2.33	
	(6.29 ± 0.03) × 10^–6^	3.00 × 10^–6^	(9.40 ± 0.35) × 10^–6^	3.77	104.0
	(6.29 ± 0.03) × 10^–6^	6.00 × 10^–6^	(1.24 ± 0.03) × 10^–5^	2.29	102.0
	(6.29 ± 0.03) × 10^–6^	9.00 × 10^–6^	(1.52 ± 0.05) × 10^–5^	3.11	99.4
**SM-WW2**	(3.15 ± 0.02) × 10^–5^		(3.18 ± 0.06) × 10^–5^	1.87	
	(3.15 ± 0.02) × 10^–5^	1.50 × 10^–5^	(4.73 ± 0.19) × 10^–5^	4.04	103.6
	(3.15 ± 0.02) × 10^–5^	3.00 × 10^–5^	(6.26 ± 0.18) × 10^–5^	2.90	102.7
	(3.15 ± 0.02) × 10^–5^	4.50 × 10^–5^	(7.88 ± 0.27) × 10^–5^	3.42	104.4

a NC: not certified; ND: not detected; RSD: relative standard deviation, SD: standard deviation.

As clearly seen, the Cu­(II) concentrations measured in the unspiked NASS-5, SM-WW1, and SM-WW2 samples were in excellent agreement with their respective certified values. For instance, the concentration found in NASS-5 was (4.63 ± 0.16) × 10^–9^ mol L^–1^, which closely matches the certified value of (4.67 ± 0.72) × 10^–9^ mol L^–1^. Similarly, the determined value for SM-WW1 was (6.28 ± 0.15) × 10^–6^ mol L^–1^ compared to the certified value of (6.29 ± 0.03) × 10^–6^ mol L^–1^, confirming the high accuracy of the proposed method.

Across all of the tested CRMs, the recovery rates for the spiked samples were highly satisfactory, ranging from 98.9 to 104.4%. The relative standard deviations (RSDs) were consistently below 4.3% (*n* = 3), indicating high precision. Furthermore, in the primary drinking water CRM, where Cu­(II) was not initially detected (ND), the spiked recoveries were similarly satisfactory (102.2–104.2%). These results clearly demonstrate that the proposed method offers high precision and reliability for the quantitative detection of Cu­(II) in various environmental water samples with minimal interference from complex matrices.

To further assess the practical applicability and reliability of the proposed method in real-world environmental samples, Cu­(II) levels were measured in groundwater, lake water, and tap water. The results from this study were statistically compared with those from a standard reference method, flame atomic absorption spectrometry (FAAS), at a 95% confidence level (*n* = 7). The analytical results and recovery data are listed in Table 2.

**Table 2:** Determination of Cu­(II) in Environmental Water Samples: A Comparison between the Proposed Method and FAAS (95% Confidence Level, *n* = 7)^a^

sample	added Cu(II) concentration (mol L^–1^)	current study (mol L^–1^)	FAAS method (mol L^–1^)	RSD (%)	recovery (%)
**ground water**		(4.60 ± 0.12) × 10^–8^	(4.65 ± 0.15) × 10^–8^		
	1.00 × 10^–7^	(1.49 ± 0.04) × 10^–7^	(1.51 ± 0.05) × 10^–7^	2.55	103.2
	2.00 × 10^–7^	(2.51 ± 0.07) × 10^–7^	(2.48 ± 0.08) × 10^–7^	2.90	102.7
	3.00 × 10^–7^	(3.53 ± 0.12) × 10^–7^	(3.55 ± 0.11) × 10^–7^	3.33	102.2
**lake water**		(1.27 ± 0.04) × 10^–7^	(1.25 ± 0.05) × 10^–7^		
	1.00 × 10^–7^	(2.29 ± 0.06) × 10^–7^	(2.30 ± 0.08) × 10^–^^7^	2.80	101.9
	2.00 × 10^–7^	(3.32 ± 0.13) × 10^–7^	(3.35 ± 0.10) × 10^–7^	3.79	102.6
	3.00 × 10^–7^	(4.37 ± 0.14) × 10^–7^	(4.32 ± 0.15) × 10^–7^	3.10	103.3
**tap water**		(1.40 ± 0.03) × 10^–7^	(1.38 ± 0.04) × 10^–7^		
	1.00 × 10^–7^	(2.43 ± 0.05) × 10^–7^	(2.41 ± 0.06) × 10^–7^	2.08	102.8
	2.00 × 10^–7^	(3.45 ± 0.07) × 10^–7^	(3.48 ± 0.09) × 10^–7^	1.94	102.4
	3.00 × 10^–7^	(4.49 ± 0.14) × 10^–7^	(4.45 ± 0.12) × 10^–7^	3.05	103.1

a RSD: relative standard deviation. The ± values represent the 95% confidence level.

As shown in the table, the initial Cu­(II) concentrations detected by the proposed method in unspiked water samples closely agreed with those obtained by FAAS. For example, the Cu­(II) concentration in groundwater was measured as (4.60 ± 0.12) × 10^–8^ mol L^–1^ using the proposed method, which is very similar to the (4.65 ± 0.15) × 10^–8^ mol L^–1^ obtained via FAAS. Similar results were observed for lake water and tap water samples, confirming the accuracy of the developed method.

Furthermore, spiking the samples with known concentrations of Cu­(II) yielded highly satisfactory recovery values, ranging from 101.9 to 103.3%, across all environmental water samples. The relative standard deviation (RSD) values were consistently low, ranging from 1.94 to 3.79%, indicating an excellent precision and reproducibility. These comparative results clearly demonstrate that the proposed method is highly accurate, free of significant matrix interference, and a reliable alternative to conventional methods for the routine monitoring of Cu­(II) in real environmental water samples.

To evaluate the analytical performance and sensitivity of the developed fluorescent probe, its Cu­(II) detection performance was systematically compared with previously reported sensors, including our recent polymeric Cu­(II) sensor and various biomass-derived carbon dots. As summarized in Table 3, the proposed pine cone-derived CD sensor exhibits a highly competitive limit of detection (LOD) of 2.8 × 10^–8^ mol L ^–1^ and a broad linear range from 2.81 × 10^–7^ to 5.87 × 10^–5^ mol L^–1^. While specific systems, such as lily bulb-derived CDs (1.28 × 10^–8^ mol L^–1^) and our previously reported polymeric sensor (2.24 × 10^–9^ mol L^–1^), achieve lower detection limits, the proposed pine cone-based sensor still outperforms several other nanomaterials, such as DNA-Au NPs and bamboo leaves-derived CDs, offering a highly practical alternative for trace level detection. These results clearly show that the synthesized carbon dots offer an excellent, sensitive, and competitive platform for the quantitative detection of Cu­(II) ions in water, particularly given their sustainable synthesis and strong matrix tolerance.

**Table 3:** Comparison of Different Methods for the Determination of Cu­(II)

source of CQDs	detection method	linear range (mol L^–1^)	LOD (mol L^–1^)	references
porfirin-PVC	fluorimetry	4.4 × 10^–6^–1 × 10^–1^	2.8 × 10^–7^	^23^
graphene oxide	fluorimetry	0–1.5 × 10^–5^	2.26 × 10^–7^	^24^
bamboo leaves	fluorimetry	3.33 × 10^–7^–6.66 × 10^–5^	1.15 × 10^–7^	^25^
boron + nitrogen	fluorimetry	1 × 10^–6^–2.5 × 10^–5^	3.0 × 10^–7^	^26^
lily bulbs	fluorimetry	5 × 10^–8^–2 × 10^–6^	1.28 × 10^–8^	^27^
petroleum coke	fluorimetry	2.5 × 10^–7^–1 × 10^–5^	2.95 × 10^–8^	^28^
DNA-Au NPs	fluorimetry	5 × 10^–7^–1 × 10^–5^	2.5 × 10^–7^	^29^
starch-stabilized Au	fluorimetry	1 × 10^–7^–1 × 10^–5^	5 × 10^–7^	^30^
DNA/Au NPs	fluorimetry	6.25 × 10^–7^–1.5 × 10^–5^	2.9 × 10^–7^	^31^
polymeric sensor	fluorimetry	7.86 × 10^–9^–1.57 × 10^–7^	2.24 × 10^–9^	^32^
pine cone	fluorimetry	2.81 × 10^–7^–5.87 × 10^–5^	2.8 × 10^–8^	this work

## 4 Conclusions

In summary, a highly sensitive, selective, and reliable fluorescence-based method was successfully developed for the quantitative determination of Cu­(II) ions in aqueous environments. The proposed method exhibited an excellent analytical performance, demonstrating strong resistance to matrix interferences. The practical applicability and accuracy of the method were rigorously validated using various certified reference materials (primary drinking water, NASS-5, SM-WW1, and SM-WW2) and real environmental water samples (groundwater, lake water, and tap water). The results yielded highly satisfactory recovery rates ranging from 98.9 to 104.4% and low relative standard deviations (RSDs of <4.3%).

Furthermore, a statistical comparison with the standard flame atomic absorption spectrometry (FAAS) method at the 95% confidence level revealed excellent agreement, confirming that the proposed method performs comparably to established conventional techniques. Given its high precision, accuracy, and excellent matrix tolerance, the developed method is a highly promising and practical alternative for the routine monitoring of trace Cu­(II) levels in environmental water samples and water quality control applications.

## Data Availability

Data used is available throughout the manuscript text.
